# Supplementary material for: Clinical usefulness of the pattern of non-adherence to anti-platelet regimen in stented patients (PARIS) thrombotic risk score to predict long-term all-cause mortality and heart failure hospitalization after percutaneous coronary intervention
Source: PLoS One. 2022 Sep 13;17(9):e0274287. doi: 10.1371/journal.pone.0274287 (PMC9469967; doi:10.1371/journal.pone.0274287)
Supplement: Attachment — Submitted filename: Response to Reviewer 2022.3.2 R1.docx [file pone.0274287.s002.docx]

**Responses to Academic Editor‘s comment**

Editor’s comment

In particular, the authors need to make a compelling argument for why the PARIS score, which was designed to asses risk of thrombotic complications after PCI, should be used to assess mortality and heart failure risk over other risk scores that were designed for this purpose. The impact of the manuscript would be improved with a formal comparison with other appropriate risk scores.

Auther’s Reply

Thank you for your helpful comment. We received a similar comment from Reviewer#2. As you pointed out, the descriptions of conventional risk scores to estimate mortality including GRACE, TIMI and CADILLAC were lacking in our original manuscript. According to your suggestion, we have revised the Introduction that firstly described the characteristics of those well-known risk scores, which could estimate the short- to mid-term outcomes of ACS patients (GRACE predicts 6 months mortality, TIMI predicts 14 days, and CADILLAC predicts 1 month and 1-year mortalities). To assess the prognostic ability of PARIS thrombotic risk score for predicting mortality, we additionally conducted the receiver operating curve analysis for PARIS thrombotic risk score and GRACE risk score in patients with ACS and compared the areas under the curves (AUC) between two scores. The AUC for predicting 6 months mortality was 0.69 in PARIS thrombotic risk score, which was comparable to 0.68 in GRACE score, as described below. The equivalence of PARIS thrombotic score to GRACE score for predicting mid-term mortality gives validity to the study, therefore we highlighted the long-term prognostic value of PARIS thrombotic risk score for not only all-cause mortality but also cardiac mortality and heart failure event, in addition to the original role for prediction of thrombotic event.

We have added the result of ROC analysis in the revised manuscript (page　34, line 336 – 339 and Fig. 4), and described about GRACE, TIMI and CADILLAC in Introduction (page 3, line55 – 60) and Discussion (page 34 – 35, line 348 – 373).

“The AUC values of the PARIS thrombotic risk score and the GRACE score for all-cause mortality in 6 months were 0.69 (95% confidence intervals 0.57–0.80) and 0.68 (95% confidence intervals 0.52–0.84). ROC analysis demonstrated that there was no significant difference between these two scores (Fig. 4).” (page 34, line 336 - 339)

Figure 4. ROC to predict all-cause mortality for 6 months in patients with acute coronary syndrome (ACS).

“Three well-known risk scores are the Global Registry of Acute Coronary Events (GRACE) score [1-3], the Thrombolysis in Myocardial Infarction (TIMI) score[4] and the Controlled Abciximab and Device Investigation to Lower Late Angioplasty Complications (CADILLIAC) score[5]. These scores combine and weigh various predictors to calculate the risk of acute coronary syndrome (ACS) for an individual patient[6].” (page 3, line55 - 60)

“The risk stratification tools for coronary artery disease after PCIs such as the GRACE score[1-3] ,the TIMI[4] and the CADILLIAC score[5] were reported to have predictive abilities for mortality in patients after PCIs. The GRACE score, which is used for overall risk assessment in patients with ACS, including ST elevation myocardial infarction (STEMI) and non-ST elevation ACS (NSTE-ACS) was designed to calculate the probability of death or myocardial infarction during admission and at 6 months by weighting 8 risk factors: age, heart rate, systolic blood pressure, initial serum creatine, Killip class, hospitalization due to cardiac arrest, positive cardiac biomarker, and ST-segment deviation[1-3, 22]. The TIMI risk score, which is often used for risk assessment in patients with NSTE-ACS, is calculated by using 7 factors: age (≥65 years); at least 3 coronary risk factors (family history, hypertension, dyslipidemia, diabetes mellitus, and smoking); known significant (≥50%) coronary stenosis; ST changes ≥ 0.5 mm on electrocardiogram; at least 2 episodes of angina in 24 hours; aspirin use in the past 7 days; and increased cardiac markers. Most of these factors can be assessed immediately after the admission. As the score increases, the incidence of major cardiovascular complications in the following 2 weeks increases synergistically[4, 22]. The CADILLIAC score, which is often used for risk assessment in patients with STEMI, evaluates several different prognostic variables including LVEF, creatinine clearance, Killip class, final TIMI flow, age, anemia and presence of three vessel disease. Utilizing the CADILLIAC risk score patients were stratified by low (CADILLIAC score 0-2), intermediate (CADILLIAC score 3-5), and high (CADILLIAC score ≥ 6) risk groups. The CADILLIAC score accurately predict 30-day and 1-year mortality after primary PCI[5].” (page 34 - 35, line 348 - 373)

We have cited the following papers as new references (#1-6, 22).

[1] Granger CB, Goldberg RJ, Dabbous O, Pieper KS, Eagle KA, Cannon CP, et al. Predictors of hospital mortality in the global registry of acute coronary events. Arch Intern Med. 2003;163:2345-53.

[2] Eagle KA, Lim MJ, Dabbous OH, Pieper KS, Goldberg RJ, Van de Werf F, et al. A validated prediction model for all forms of acute coronary syndrome: estimating the risk of 6-month postdischarge death in an international registry. Jama. 2004;291:2727-33.

[3] Fox KA, Dabbous OH, Goldberg RJ, Pieper KS, Eagle KA, Van de Werf F, et al. Prediction of risk of death and myocardial infarction in the six months after presentation with acute coronary syndrome: prospective multinational observational study (GRACE). Bmj. 2006;333:1091.

[4] Antman EM, Cohen M, Bernink PJ, McCabe CH, Horacek T, Papuchis G, et al. The TIMI risk score for unstable angina/non-ST elevation MI: A method for prognostication and therapeutic decision making. Jama. 2000;284:835-42.

[5] Halkin A, Singh M, Nikolsky E, Grines CL, Tcheng JE, Garcia E, et al. Prediction of mortality after primary percutaneous coronary intervention for acute myocardial infarction: the CADILLAC risk score. J Am Coll Cardiol. 2005;45:1397-405.

[6] Poldervaart JM, Langedijk M, Backus BE, Dekker IMC, Six AJ, Doevendans PA, et al. Comparison of the GRACE, HEART and TIMI score to predict major adverse cardiac events in chest pain patients at the emergency department. Int J Cardiol. 2017;227:656-61.

[22] Kimura K, Kimura T, Ishihara M, Nakagawa Y, Nakao K, Miyauchi K, et al. JCS 2018 Guideline on Diagnosis and Treatment of Acute Coronary Syndrome. Circ J. 2019;83:1085-196.

**Responses to Reviewer #1**

We appreciate your detailed reviews on our manuscript. We have revised our manuscript according to your comments.

Reviewer’s comment

That being said however the study is underpowered being that it is single centered and with few patients. Also as the authors have pointed out that nearly 80% of it study subjects are men. And being that these patients are from 2010-2018, there are certain caveats when applying any risk score that does take into account angiographic findings such as how the PCIs were done i.e. use of imaging and physiologic guidance, stent variety and generation, taking into account the usage of DCBs which are not applicable to coronaries in the US, the ~10% use of BMS, etc. Also of note and as mentioned by the authors in the lines 312 -315 variables only related to the hospitalization were taken into account for. Thus the large part of our therapy outside of PCI as related to medication optimization with goal directed dosage levels was not addressed with this study. The tables were extensive and had multiple different characteristics that were compiled. However, these issues we believe are minor and more critique of the content rather than any major issues.

Author’s Reply

Thank you for your important comments. As you mentioned, 78.6% of patients in our study are men, which may reflect the true prevalence in Japanese population of patients with coronary artery disease. The other large registries of coronary artery disease patients who underwent percutaneous coronary intervention (PCI) in Japan showed that 78.3% were male in PENDULUM registry [Nakamura M, Kadota K, Nakao K, Nakagawa Y, Shite J, Yokoi H, et al. High bleeding risk and clinical outcomes in East Asian patients undergoing percutaneous coronary intervention: the PENDULUM registry. *EuroIntervention* 2021; 19: 1154-1162] and 73.0% were male in CREDO-Kyoto registry [Application of the Modified High Bleeding Risk Criteria for Japanese Patients in an All-Comers Registry of Percutaneous Coronary Intervention -From the CREDO-Kyoto Registry Cohort-3-. *Circ J* 2021; 85: 769-781]. The duration of enrollment in this study was relatively long, from 2010 to 2018, thus there might be some differences in strategies for treatment, as you mentioned. Hence, we have analyzed the proportion of use of imaging devices during PCI, which was over 95% in all three groups as additionally displayed in Table 1, so that showing the consistent imaging device-guided PCI was performed during all periods. However, unfortunately, data of stent generation and goal directed dosage levels of medication were not available, though those were very important information for understanding the patient background and relationship to the outcomes, as you pointed out.

Therefore, we have corrected the limitations as follow, reflecting your comments and also suggestions from Reviewer #2.

“The present study had several limitations. First, as a retrospective cohort study, the study may be with inherent limitation to selection and ascertainment biases. Second, clinical practices in PCI and medical treatment in the study may have some differences in the setting of each period. Third, it may be difficult to determine the reproducibility and generalizability of PARIS thrombotic risk score to new patient cohort because of the absence of external validation of this study. Fourth, we used only variables on hospitalization in this study, without taking into consideration changes in medical parameters and post discharge treatment.” (page 37, lines 421 - 428).

Reviewer’s comment

Minor Issues are grammatical in lines 48-53 would suggest to better copyedit this part "...and has developed (into) one of the most popular treatment(s) in modern cardiovascular disease. It is necessary for (would take out "the") patients who under(go) PCI (no plural s required)..."

Author’s Reply

We apologize for those mistakes and have corrected those mistakes in page 3, lines 52 - 53.

Reviewer’s comment

Lastly also a minor issue in our opinion there is a lack of discussion regarding the findings of cardiac death in the Low, Intermediate, High Risk PARIS score patients as described in Graph B of Figure 1. The focus as it rightly should be on all-cause mortality and CHF events. It would be ideal and interesting to discuss the findings of cardiac death in particular perhaps why these grafts initially partial converge and then after one sees the high risk patients diverge from the intermediate and low risk patients around the ~800 days mark.

Author’s Reply

Thank you for your helpful comment. The 64 cardiac deaths included 48 deaths from heart failure, therefore, the majority of cardiac deaths were heart failure death. Unfortunately, we could not fully explain the reason why the Kaplan-Meier curves for cardiac mortality initially partial converge and the high-risk group diverged from other two groups around 800 days. The Kaplan-Meier curves for hospitalization due to heart failure also initially partial converge and the high-risk group diverged from other two groups around 800 days. The worsening of heart failure with aging may be one of the reasons for the results that you pointed out. We have added the following sentences about these issues in page 35 - 36, lines 381 - 386.

**“**The reason why the Kaplan-Meier curves for cardiac mortality and hospitalization due to heart failure partial converge and the high-risk group diverged from other two groups around 800 days is not unclear. One possible reason is the 64 cardiac deaths included 48 deaths from heart failure, therefore, the majority of cardiac deaths were heart failure death. The worsening of heart failure with aging may be one of the reasons for the results.”

**Thank you again for your invaluable comments to our manuscript.**

**Responses to Reviewer #2**

We appreciate your detailed reviews on our manuscript. We have revised our manuscript according to your comments.

Reviewer’s comment

1- Study rationale and hypothesis: The authors chose to calculate the PARIS score for all subjects and present a study to justify its prognostic utility for long term outcomes. While the study population, follow-up duration, comprehensiveness of data and event rate is suitable for such goal, the choice of PARIS score over other available and widely used score for ACS and PCI such as GRACE, TIMI, CADILLAC, among others is not well justified in the introduction or the discussion. The authors highlighted the strengths of their study in comparison to those done by others in the discussion, but it is not true that GRACE and TIMI were not studied for longer term outcomes. Further, the PARIS score albeit simple and easy to use yet is limited with choice of variables (no age, gender, and co-morbidities such as anemia included in the score). Hence, in order to give validity to their study, I most certainly recommend comparing the prognostic value of PARIS score in this population against GRACE and TIMI score and see if it outperforms either one. By doing so, you will give your study more clinical meaning and strength to your chosen score.

Author’s Reply

Thank you for your helpful comment. As you pointed out, the descriptions of conventional risk scores including GRACE, TIMI and CADILLAC were lacking in our original manuscript. According to your suggestion, we have revised the Introduction that firstly described the characteristics of those well-known risk scores, which could estimate the short- to mid-term outcomes of ACS patients (GRACE predicts 6 months mortality, TIMI predicts 14 days and CADILLAC predicts 1 month and 1-year mortalities). To assess the prognostic ability of PARIS thrombotic risk score for predicting mortality, we additionally conducted the receiver operating curve analysis for PARIS thrombotic risk score and GRACE risk score in patients with ACS and compared the areas under the curves (AUC) between two scores, according to your recommendation. The AUC for predicting 6 months mortality was 0.69 in PARIS thrombotic risk score, which was comparable to 0.68 in GRACE score, as described below. Unfortunately, the comparing of PARIS thrombotic risk score to TIMI risk score could not be performed, since the data for calculating TIMI risk score were not available in this cohort. The equivalence of PARIS thrombotic score to GRACE score for predicting mid-term mortality gives validity to the study, therefore we highlighted the long-term prognostic value of PARIS thrombotic risk score for not only all-cause mortality but also cardiac mortality and heart failure event, in addition to the original role for prediction of thrombotic event. We have added the result of ROC analysis in the revised manuscript (page　34, line 336 – 339 and Fig. 4), and described about GRACE, TIMI and CADILLAC in Introduction (page 3, line 55 - 60) and Discussion (page 34 - 35, line 348 - 373).

“The AUC values of the PARIS thrombotic risk score and the GRACE score for all-cause mortality in 6 months were 0.69 (95% confidence intervals 0.57–0.80) and 0.68 (95% confidence intervals 0.52–0.84). ROC analysis demonstrated that there was no significant difference between these two scores (Fig. 4).” (page 34, line 336 – 339)

Figure 4. ROC to predict all-cause mortality for 6 months in patients with acute coronary syndrome (ACS).

“Three well-known risk scores are the Global Registry of Acute Coronary Events (GRACE) score [1-3], the Thrombolysis in Myocardial Infarction (TIMI) score[4] and the Controlled Abciximab and Device Investigation to Lower Late Angioplasty Complications (CADILLIAC) score[5]. These scores combine and weigh various predictors to calculate the risk of acute coronary syndrome (ACS) for an individual patient[6].” (page 3, line 55 - 60)

“The risk stratification tools for coronary artery disease after PCIs such as the GRACE score[1-3] ,the TIMI[4] and the CADILLIAC score[5] were reported to have predictive abilities for mortality in patients after PCIs. The GRACE score, which is used for overall risk assessment in patients with ACS, including ST elevation myocardial infarction (STEMI) and non-ST elevation ACS (NSTE-ACS) was designed to calculate the probability of death or myocardial infarction during admission and at 6 months by weighting 8 risk factors: age, heart rate, systolic blood pressure, initial serum creatine, Killip class, hospitalization due to cardiac arrest, positive cardiac biomarker, and ST-segment deviation[1-3, 22]. The TIMI risk score, which is often used for risk assessment in patients with NSTE-ACS, is calculated by using 7 factors: age (≥65 years); at least 3 coronary risk factors (family history, hypertension, dyslipidemia, diabetes mellitus, and smoking); known significant (≥50%) coronary stenosis; ST changes ≥ 0.5 mm on electrocardiogram; at least 2 episodes of angina in 24 hours; aspirin use in the past 7 days; and increased cardiac markers. Most of these factors can be assessed immediately after the admission. As the score increases, the incidence of major cardiovascular complications in the following 2 weeks increases synergistically[4, 22]. The CADILLIAC score, which is often used for risk assessment in patients with STEMI, evaluates several different prognostic variables including LVEF, creatinine clearance, Killip class, final TIMI flow, age, anemia and presence of three vessel disease. Utilizing the CADILLIAC risk score patients were stratified by low (CADILLIAC score 0-2), intermediate (CADILLIAC score 3-5), and high (CADILLIAC score ≥ 6) risk groups. The CADILLIAC score accurately predict 30-day and 1-year mortality after primary PCI[5].” (page 34 - 35, line 348 - 373)

We have cited the following papers as new references (#1-6, 22).

[1] Granger CB, Goldberg RJ, Dabbous O, Pieper KS, Eagle KA, Cannon CP, et al. Predictors of hospital mortality in the global registry of acute coronary events. Arch Intern Med. 2003;163:2345-53.

[2] Eagle KA, Lim MJ, Dabbous OH, Pieper KS, Goldberg RJ, Van de Werf F, et al. A validated prediction model for all forms of acute coronary syndrome: estimating the risk of 6-month postdischarge death in an international registry. Jama. 2004;291:2727-33.

[3] Fox KA, Dabbous OH, Goldberg RJ, Pieper KS, Eagle KA, Van de Werf F, et al. Prediction of risk of death and myocardial infarction in the six months after presentation with acute coronary syndrome: prospective multinational observational study (GRACE). Bmj. 2006;333:1091.

[4] Antman EM, Cohen M, Bernink PJ, McCabe CH, Horacek T, Papuchis G, et al. The TIMI risk score for unstable angina/non-ST elevation MI: A method for prognostication and therapeutic decision making. Jama. 2000;284:835-42.

[5] Halkin A, Singh M, Nikolsky E, Grines CL, Tcheng JE, Garcia E, et al. Prediction of mortality after primary percutaneous coronary intervention for acute myocardial infarction: the CADILLAC risk score. J Am Coll Cardiol. 2005;45:1397-405.

[6] Poldervaart JM, Langedijk M, Backus BE, Dekker IMC, Six AJ, Doevendans PA, et al. Comparison of the GRACE, HEART and TIMI score to predict major adverse cardiac events in chest pain patients at the emergency department. Int J Cardiol. 2017;227:656-61.

[22] Kimura K, Kimura T, Ishihara M, Nakagawa Y, Nakao K, Miyauchi K, et al. JCS 2018 Guideline on Diagnosis and Treatment of Acute Coronary Syndrome. Circ J. 2019;83:1085-196.

Reviewer’s comment

2- Study design and methodology: This is a retrospective cohort study not a prospective one as highlighted in the limitations section. The authors nicely outlined all the steps they undertook to ascertain their outcomes and all the clinical variables of the study, all of which are strengths that can be highlighted in the limitation section.
Recommendations:
- However, there has to be data provided on (1) the type of ACS (STEMI, NSTEMI, UA) and (2) any exclusion criteria (did you include consecutive patients, exclude those with unavailable data on follow-up or STEMI, etc.).

Author’s Reply

Thank you for your helpful comments.　We have showed the type of ACS in Table 1 and added exclusion criteria in page 4, line 88.

“Exclusion criteria were defined as death at discharge.”

Reviewer’s comment
- Give the presence of competing risk between HF hospitalization and cardiac mortality, the authors are urged to present competing risks survival analysis in their Cox regression for the HF hospitalization and cardiac mortality outcomes.

Author’s Reply

Thank you for pointing out this important issue. We have investigated cardiac mortality and hospitalization due to heart failure by cumulative incidence competing risk method. The cumulative incidence curves have revealed that hospitalization due to heart failure after adjusting competing risks was highest in high-risk group among the three groups as shown Figure 2(B). Furthermore, results from the Fine and Gray model (hazard of the subdistribution model) of association of PARIS thrombosis risk score with cardiac mortality and hospitalization due to heart failure were presented in Tables 6-8. We have added the result of cumulative incidence competing risk method and Fine and Gray model in the revised manuscript (page　15, line 210 – 212 and page 26 – 32, line 276 - 327).

“In cumulative incidence competing risk method (Fig 2), the hospitalization due to heart failure after adjusting competing risk was highest in high-risk group among the 3 groups (P < 0.001).” (page 15, line 210 – 212)

Figure 2. The cumulative incidence curves for cardiac mortality (A) and hospitalization due to heart failure (B) in the high, intermediate, and low PARIS thrombotic risk score groups.

“**Fine and Gray model (hazard of the subdistribution model)**

The Fine and Gray model (hazard of the subdistribution model) of association of the PARIS thrombotic score, which are presented as categorical variables (groups), with cardiac mortalities and hospitalization due to heart failure are presented in Tables 6–7.”

“Moreover, we performed the Fine and Gray model (hazard of the subdistribution model) of association of the PARIS thrombotic risk score, which are presented as continuous variables (per 1-point increase), with cardiac mortalities and hospitalization due to heart failure as sensitivity analyses (Table 8). In the subdistribution multivariable models, a high PARIS thrombotic score was determined to be an independent predictor of hospitalization due to heart failure after adjusting for other confounding factors (hazard ratio 1.28 per 1-point increase, 95% confidence intervals 1.10–1.50, P = 0.001).” (page 26 – 32, line 276 - 327)

**Table 6. Fine and Gray model (hazard of the subdistribution model) for cardiac mortality**

| Variable | Univariate　analysis | | | Multivariate analysis^#^ | | |
| --- | --- | --- | --- | --- | --- | --- |
|  | sHR | 95% Cl | *P* value | sHR | 95% Cl | *P* value |
| PARIS thrombotic score (low-risk group) | Ref. |  |  | Ref. |  |  |
| Intermediate-risk group (vs. low-risk group) | 1.50 | 0.79 – 2.85 | 0.210 | 1.78 | 0.83 – 3.81 | 0.130 |
| High-risk group (vs. low-risk group) | 2.07 | 1.06 – 4.04 | 0.032 | 1.49 | 0.66 – 3.32 | 0.330 |
| Age (per 1year increase) | 1.04 | 1.01 – 1.06 | 0.002 | 1.02 | 1.00 – 1.05 | 0.050 |
| Male | 0.95 | 0.53 – 1.71 | 0.890 |  |  |  |
| Smoking | 1.23 | 0.71 – 2.13 | 0.460 |  |  |  |
| History of PCI | 1.27 | 0.71 – 2.29 | 0.410 |  |  |  |
| History of CABG | 2.44 | 0.98 – 6.06 | 0.054 |  |  |  |
| History of OMI | 0.97 | 0.42 – 2.22 | 0.960 |  |  |  |
| Diabetes mellitus | 1.16 | 0.70 – 1.93 | 0.540 |  |  |  |
| Anemia | 3.01 | 1.72 – 5.27 | <0.001 | 1.83 | 1.02 – 3.28 | 0.042 |
| Chronic kidney disease | 2.86 | 1.68 – 4.88 | <0.001 |  |  |  |
| Dialysis | 3.36 | 1.70 – 6.61 | <0.001 |  |  |  |
| Peripheral artery disease | 1.84 | 1.00 – 3.38 | 0.047 | 1.22 | 0.63 – 2.36 | 0.550 |
| Atrial fibrillation | 2.19 | 1.26 – 3.82 | 0.005 | 1.63 | 0.93 – 2.83 | 0.084 |
| Acute coronary syndrome | 0.83 | 0.51 – 1.34 | 0.450 |  |  |  |
| Multivessel disease | 2.25 | 1.34 – 3.76 | 0.002 | 1.96 | 1.08 – 3.56 | 0.027 |
| Target lesion (RCA） | 0.62 | 0.35 – 1.09 | 0.098 |  |  |  |
| Target lesion (LAD) | 0.93 | 0.57 – 1.51 | 0.800 |  |  |  |
| Target lesion (LCX) | 1.67 | 0.96 – 2.91 | 0.065 |  |  |  |
| Target lesion (LMT) | 2.04 | 0.73 – 5.70 | 0.170 |  |  |  |
| LVEF (per 1% increase) | 0.94 | 0.93 – 0.96 | <0.001 | 0.95 | 0.94 – 0.97 | <0.001 |
| BMS | 0.33 | 0.12 – 0.95 | 0.041 | 0.40 | 0.13 – 1.20 | 0.100 |
| DES | 1.18 | 0.66 – 2.12 | 0.560 |  |  |  |
| β-blockers | 2.36 | 1.26 – 4.42 | 0.007 | 1.69 | 0.86 – 3.30 | 0.130 |
| RAS inhibitors | 1.24 | 0.66 – 2.34 | 0.500 |  |  |  |
| Proton pump inhibitor | 1.52 | 0.83 – 2.77 | 0.170 |  |  |  |

#. In the multivariate analysis, PARIS thrombotic score was forcedly entered, and thereafter adjusted for age, anemia, peripheral artery disease, atrial fibrillation, multivessel disease, LVEF, BMS and β-blockers with the forced entry method.

sHR, subdistribution hazard ratio; PARIS, the patterns of non-adherence to anti-platelet regimen in stented patients; PCI, percutaneous coronary intervention; CABG, coronary artery bypass grafting; OMI, old myocardial infarction; RCA, right coronary artery; LAD, left anterior descending coronary artery; LCX, left circumflex coronary artery LMT, left main trunk; LVEF, left ventricular ejection fraction; BMS, bare metal stent; DES, drug eluting stent; RAS, Renin-Angiotensin-Aldosterone System.

**Table 7. Fine and Gray model (hazard of the subdistribution model) for hospitalization due to heart failure**

| Variable | Univariate　analysis | | | Multivariate analysis^#^ | | |
| --- | --- | --- | --- | --- | --- | --- |
|  | sHR | 95% Cl | *P* value | sHR | 95% Cl | *P* value |
| PARIS thrombotic score (low-risk group) | Ref. |  |  | Ref. |  |  |
| Intermediate-risk group (vs. low-risk group) | 1.18 | 0.51 – 2.72 | 0.700 | 0.99 | 0.42 – 2.32 | 0.980 |
| High-risk group (vs. low-risk group) | 3.23 | 1.47 – 7.09 | 0.003 | 2.10 | 0.89 – 4.95 | 0.088 |
| Age (per 1year increase) | 1.01 | 0.98 – 1.04 | 0.330 |  |  |  |
| Male | 0.52 | 0.29 – 0.91 | 0.022 | 0.55 | 0.30 – 1.00 | 0.051 |
| Smoking | 0.82 | 0.47 – 1.44 | 0.500 |  |  |  |
| History of PCI | 1.40 | 0.75 – 2.60 | 0.280 |  |  |  |
| History of CABG | 3.16 | 1.31 – 7.63 | 0.010 |  |  |  |
| History of OMI | 1.38 | 0.66 – 2.89 | 0.380 |  |  |  |
| Diabetes mellitus | 1.78 | 0.99 – 3.20 | 0.054 |  |  |  |
| Anemia | 2.28 | 1.25 – 4.16 | 0.006 | 1.40 | 0.74 – 2.64 | 0.300 |
| Chronic kidney disease | 3.85 | 2.02 – 7.33 | <0.001 |  |  |  |
| Dialysis | 0.80 | 0.27 – 2.37 | 0.690 |  |  |  |
| Peripheral artery disease | 2.06 | 1.09 – 3.89 | 0.025 | 1.23 | 0.62 – 2.41 | 0.550 |
| Atrial fibrillation | 3.12 | 1.82 – 5.35 | <0.001 | 2.37 | 1.34 – 4.19 | 0.003 |
| Acute coronary syndrome | 1.07 | 0.62 – 1.85 | 0.780 |  |  |  |
| Multivessel disease | 1.57 | 0.89 – 2.79 | 0.120 |  |  |  |
| Target lesion (RCA） | 0.92 | 0.51 – 1.65 | 0.800 |  |  |  |
| Target lesion (LAD) | 1.24 | 0.72 – 2.14 | 0.430 |  |  |  |
| Target lesion (LCX) | 0.56 | 0.22 – 1.38 | 0.210 |  |  |  |
| Target lesion (LMT) | 1.77 | 0.42 – 7.34 | 0.430 |  |  |  |
| LVEF (per 1% increase) | 0.96 | 0.95 – 0.96 | <0.001 | 0.98 | 0.96 – 0.99 | 0.044 |
| BMS | 0.31 | 0.11 – 0.84 | 0.021 | 0.66 | 0.18 – 2.38 | 0.530 |
| DES | 2.18 | 1.10 – 4.34 | 0.026 | 1.69 | 0.66 – 4.37 | 0.270 |
| β-blockers | 2.28 | 1.10 – 4.72 | 0.026 | 1.48 | 0.70 – 3.13 | 0.300 |
| RAS inhibitors | 1.33 | 0.65 – 2.73 | 0.420 |  |  |  |
| Proton pump inhibitor | 2.05 | 1.06 – 3.98 | 0.032 | 1.32 | 0.69 – 2.52 | 0.390 |

#. In the multivariate analysis, PARIS thrombotic score was forcedly entered, and thereafter adjusted for male, anemia, peripheral artery disease, atrial fibrillation, LVEF, BMS, DES, β-blockers and proton pump inhibitor with the forced entry method.

sHR, subdistribution hazard ratio; PARIS, the patterns of non-adherence to anti-platelet regimen in stented patients; PCI, percutaneous coronary intervention; CABG, coronary artery bypass grafting; OMI, old myocardial infarction; RCA, right coronary artery; LAD, left anterior descending coronary artery; LCX, left circumflex coronary artery LMT, left main trunk; LVEF, left ventricular ejection fraction; BMS, bare metal stent; DES, drug eluting stent; RAS, Renin-Angiotensin-Aldosterone System.

**Table 8. Fine and Gray model (hazard of the subdistribution model) of PARIS thrombotic score (continuous variables) for cardiac mortalities and hospitalization due to heart failure**

| Variable | Univariate　analysis | | | Multivariate analysis | | |
| --- | --- | --- | --- | --- | --- | --- |
|  | sHR | 95% Cl | *P* value | sHR | 95% Cl | *P* value |
| **Cardiac mortality** |  |  |  |  |  |  |
| PARIS thrombotic score (per 1-point increase) ^#1^ | 1.17 | 1.03 – 1.33 | 0.014 | 1.08 | 0.95 – 1.23 | 0.210 |
| **Hospitalization due to heart failure** |  |  |  |  |  |  |
| PARIS thrombotic score (per 1-point increase) ^#2^ | 1.36 | 1.19 – 1.56 | <0.001 | 1.28 | 1.10 – 1.50 | 0.001 |

#1. In the multivariate analysis, PARIS thrombotic score was forcedly entered, and thereafter adjusted for age, anemia, peripheral artery disease, atrial fibrillation, multivessel disease, LVEF, BMS and β-blockers with the forced entry method.

#2. In the multivariate analysis, PARIS thrombotic score was forcedly entered, and thereafter adjusted for male, anemia, peripheral artery disease, atrial fibrillation, LVEF, BMS, DES, β-blockers and proton pump inhibitor with the forced entry method.

sHR, subdistribution hazard ration

Reviewer’s comment
- I recommend to do a stratified analysis on the basis on presentation (ACS vs stable angina) as it is important to see if the PARIS score's prognostic potential holds for either presentation. Comparing the score with GRACE and TIMI is invalid for those with stable angina.

Author’s Reply

Thank you for your important comment. We have conducted subgroup analysis to assess the prognostic value of the PARIS thrombosis risk score for all-cause and cardiac mortality and incidence of hospitalization due to heart failure by clinical presentations. There was no interaction between ACS and stable AP. We have added this issue in page 15 - 16, line 223 – 235 and Figure 3.

“Furthermore, to assess the prognostic value of the PARIS thrombosis risk score for all-cause and cardiac mortality and incidence of hospitalization due to heart failure by clinical presentations, we conducted subgroup analysis (Fig. 3). Subgroup analysis was performed to compare the high-risk group with the low-risk group. The hazard ratios of the PARIS thrombotic risk score on all-cause mortality in patients presenting stable angina pectoris and acute coronary syndrome were 1.72 (95% confidence intervals 1.01–2.93) and 3.30 (95% confidence intervals 1.63–6.67). The hazard ratios of the PARIS thrombotic risk score on cardiac mortality in patients presenting stable angina pectoris and acute coronary syndrome were 1.55 (95% confidence intervals 0.62–3.84) and 7.92 (95% confidence intervals 1.05–59.80). The hazard ratios of the PARIS thrombotic risk score on the incidence of hospitalization due to heart failure in patients presenting stable angina and acute coronary syndrome were 3.66 (95% confidence intervals 1.69–7.90) and 4.99 (95% confidence intervals 1.53–16.29). There was no interaction.” (page 15 - 16, line 223 – 235)

Figure 3. Forest Plot of hazard ratios by clinical presentations.

Reviewer’s comment
3- Limitations: The biggest limitation of the study is not the small sample size as authors mentioned but (1) retrospective design with inherent limitation to selection and ascertainment biases, (2) the absence of external validation of the proposed score, (3) absence of comparison of PARIS score with other widely used and validated risk scores such as GRACE and TIMI.

Author’s Reply

Thank you for your important comments. We have corrected the limitations as below;

“The present study had several limitations. First, as a retrospective cohort study, the study may be with inherent limitation to selection and ascertainment biases. Second, clinical practices in PCI and medical treatment in the study may have some difference in the setting of each period. Third, it may be difficult to determine the reproducibility and generalizability of PARIS thrombotic risk score to new patient cohort because of the absence of external validation of this study. Fourth, we used only variables on hospitalization in this study, without taking into consideration changes in medical parameters and post discharge treatment.” (page 37, line 421 - 428).

Regarding the absence of comparison of PARIS thrombotic risk score with other risk score, we have described as above.

Reviewer’s comment

4- Language, technical terms and design: The authors need to improve the writing of the paper in terms of English language, particularly in the introduction. The words "dependable" and "helpful" to describe PCI are not appropriate, and repeating the aims statement with the previous sentence of what's missing in the literature (i.e. the long-term prognostic use of PARIS score) is also not needed. I suggest getting the help of a native English language speaker to proofread the paper.
Further, I could not fully read the tables presented as they were embedded in the text and truncated on the right, making half the tables missing to me. The KM curves are also of very poor quality and need to be presented in a higher resolution.

Author’s Reply

We apologize for mistakes of English language and careless of tables and figures. We have corrected “dependable and helpful” to “well-established” in page 3, line 55. In revised manuscript, you can read fully tables and high-resolution KM curves.

**Thank you again for your invaluable comments to our manuscript.**
