# Supplementary material for: Clinical usefulness of the pattern of non-adherence to anti-platelet regimen in stented patients (PARIS) thrombotic risk score to predict long-term all-cause mortality and heart failure hospitalization after percutaneous coronary intervention
Source: PLoS One. 2022 Sep 13;17(9):e0274287. doi: 10.1371/journal.pone.0274287 (PMC9469967; doi:10.1371/journal.pone.0274287)
Supplement: Attachment — Submitted filename: Response to Reviewer R2 2022.5.29.docx [file pone.0274287.s003.docx]

**Responses to Academic Editor‘s comment**

**Editor’s comment**

**In addition to the points raised by Reviewer #2, the Editor asks that you review the manuscript for grammatical integrity. For example, there are several places where single sentences serve as stand-alone paragraphs. These should be incorporated into adjacent paragraphs.**

Author’s Reply

*Thank you for your helpful comment.* *We have found two places where single sentences server as stand-alone paragraphs (page 3, lines 62–64 and page 40, lines 474–475 in the revised manuscript with track changes). We have removed these sentences, as shown below, because they had previously caused some difficulties with regard to following the manuscript. Accordingly, the reference [7] as shown below has been removed. Additionally, we have asked the native English speaker to proofread and have also been modified to make the manuscript easier to understand and to correct any previous grammatical errors as described in “the revised manuscript with track changes”.*

“Moreover, it is necessary for patients who undergo PCI or stent implantation to take a dual antiplatelet therapy (DAPT) with aspirin and a P2Y12 receptor inhibitor in order to prevent serious stent-related thrombotic complications after PCIs [7].” (page 3, lines 62–64)

“Findings in this study indicate that the PARIS thrombotic risk score may be used clinically beyond its intended use.” (page 40, lines 474–475)

“[7] Mauri L, Kereiakes DJ, Yeh RW, Driscoll-Shempp P, Cutlip DE, Steg PG, et al. Twelve or 30 months of dual antiplatelet therapy after drug-eluting stents. N Engl J Med. 2014;371:2155-66.“

*The following reference has been deleted because the content is supplemented by reference [17].*

[18] Ponikowski P, Voors AA, Anker SD, Bueno H, Cleland JGF, Coats AJS, et al. 2016 ESC Guidelines for the diagnosis and treatment of acute and chronic heart failure: The Task Force for the diagnosis and treatment of acute and chronic heart failure of the European Society of Cardiology (ESC)Developed with the special contribution of the Heart Failure Association (HFA) of the ESC. Eur Heart J. 2016;37:2129-200.

*Reference [15] has been added to correct reference [16].*

[15] Correction: Using standardized serum creatinine values in the Modification of Diet in Renal Disease Study Equation. Ann Intern Med. 2021;174:584.

*The following references have been delated as the manuscript has been revised.*

[23] Tang EW, Wong CK, Herbison P. Global Registry of Acute Coronary Events (GRACE) hospital discharge risk score accurately predicts long-term mortality post acute coronary syndrome. Am Heart J. 2007;153:29-35.

[24] Valgimigli M, Bueno H, Byrne RA, Collet JP, Costa F, Jeppsson A, et al. 2017 ESC focused update on dual antiplatelet therapy in coronary artery disease developed in collaboration with EACTS: The Task Force for dual antiplatelet therapy in coronary artery disease of the European Society of Cardiology (ESC) and of the European Association for Cardio-Thoracic Surgery (EACTS). Eur Heart J. 2018;39:213-60.

[25] Tanaka S, Sakata R, Marui A, Furukawa Y, Kita T, Kimura T. Predicting long-term mortality after first coronary revascularization: – the Kyoto model –. Circ J. 2012;76:328-34.

[26] Nammas W, Kiviniemi T, Schlitt A, Rubboli A, Valencia J, Lip GYH, et al. Value of DAPT score to predict adverse outcome in patients with atrial fibrillation undergoing percutaneous coronary intervention: A post-hoc analysis from the AFCAS registry. Int J Cardiol. 2018;253:35-9.

**Responses to Reviewer #2**

*We appreciate your detailed review of our manuscript. We believe we have revised the manuscript accordingly.*

**Reviewer’s comment**

**The authors did an excellent job responding to all the comments raised, particularly ones related to the scientific rationale and statistical analysis of the data. I have no follow up comments to my previously raised one.
However, I do think that the data presented are elegantly tabulated and charted and deserve a better discussion.**

**The only major comment I currently have is that there is needs to be major improvement to the discussion section in terms of writeup. It is really hard to follow, with no clear flow of ideas of objective, a lot of redundancy and unnecessary details, and relatively weak critical thinking and reasoning of the data. Here are my suggestions:
1- You need to show why is your data important relative to prior studies (one in particular you cited by Zhao et al.). You need to go in detail about their patient population, how they validated their score against other ones, what makes you study better, etc. (you can't just say longer term follow up).**

Author’s Reply

*Thank you for your helpful suggestions. We have described in detail about the differences between our study and the prior one. We have addressed this issue in the Discussion (page 36, line 391–page 37, line 403).*

“A few studies have reported on the association between PARIS thrombotic risk score and mortality in patients with PCIs. Zhao et al. reported that the PARIS thrombotic risk score was a significant independent predictor of 2-year mortality in patients after PCIs [26]. One of the advantages of the present study is that our follow-up period was much longer than that in Zhao et al.’s report. Their study population only included patients who had undergone drug eluting stent (DES) implantation, whereas the present study also included patients who had not undergone such implantation, which is useful in terms of stent-less PCI. Moreover, their study did not include a multivariate analysis in assessing the prognostic value of the PARIS thrombotic risk score for all-cause mortality. In contrast, in the present study we performed multivariate Cox proportional hazard analysis followed by competing risk analysis to assess the prognostic value of the PARIS thrombotic risk score. Therefore, the present study provides a new insight into the clinical usefulness of the PARIS thrombotic risk score.” (page 36, line 391–page 37, line 403)

**Reviewer’s comment**

**2- Avoid redundancy. Every paragraph end by "we show that PARIS score predict outcomes...predict mortality...etc.". You need to put more thought into how your score can be used in the clinical setting (for example, can be used preoperatively, postop to allow for shared decision making and prevent HF hospitalization, etc.).**

Author’s Reply

*According to your comment, we have added the following sentences, which include more insight how the PARIS score can be used in the clinical setting (page 36, lines 383–390).*

“Heart failure remains a major cause of mortality, morbidity, hospitalization, and poor quality of life in patients with coronary artery disease [25], thus identifying patients at risk of heart failure is important to improve their prognosis after PCIs. The PARIS thrombotic risk score can be used to allow for shared decision making and prevent hospitalization due to heart failure pre- and post-operatively. We believe that the results of the current study will help clinicians to identify high-risk patients and to plan early examinations and interventions.” (page 36, lines 383–390)

*Regarding avoiding redundancy, please see our next reply.*

**Reviewer’s comment**

**3- Need to have stronger arguments and better flow of paragraphs. Each paragraph needs to be linked to the one before, start and end with a compelling statement about a certain finding, and end with a concluding statement about the following paragraph or future applications/outlook of your findings.**

Author’s Reply

*We have reconsidered the flow of paragraphs and reconstructed the discussion. At first, we modified* “However, these risk scores were estimated for predicting short to mid-term prognosis, thus the long-term follow-up after PCI were poorly documented[23]. Therefore, the present study has the advantages of enrolling all patients who underwent PCI and may aid in predicting long-term prognosis after PCI.” (page 37, lines 395–399 in the revised manuscript with track changes) *to* “However, there have been few risk scores that include patients with not only ACS, but also chronic coronary syndrome, and can be used to predict long term mortalities. The ability of PARIS thrombotic risk score to predict the 6-month mortality of patients with ACS was comparable to that of the GRACE score; hence, we examined the long-term prognostic value of PARIS thrombotic risk score for both ACS and chronic coronary syndrome” (page 35, lines 368–373).

*Moreover, the following sentences have been removed to avoid redundancy and be linked to the one before, start and end with a compelling statement about a certain finding, and end with a concluding statement about the following paragraph or future applications/outlook of our findings.*

“The results of this study … improve the prognosis after PCI.” (page 38, line 405–page 39, line 432 in the revised manuscript with track changes)

“Findings in this study indicate that the PARIS thrombotic risk score may be used clinically beyond its intended use.” (page 40, line 474–page 41, line 475 in the revised manuscript with track changes)

*Moreover, we have added the sentences below to achieve the same purpose.*

“Heart failure remains a major cause of mortality, morbidity, hospitalization, and poor quality of life in patients with coronary artery disease [25]; thus, identifying patients at risk of heart failure is important to improve their prognosis after PCIs. The PARIS thrombotic risk score can be used to allow for shared decision making and prevent hospitalization due to heart failure pre- and postoperatively. We believe that the results of the current study will help clinicians to identify high-risk patients and to plan early examinations and interventions.” (page 36, lines 383–390)

*The text below has been repositioned on page 37, lines 411–416 to improve the flow of paragraphs.*

“The reason why the Kaplan-Meier curves for cardiac mortality and hospitalization due to heart failure partially converge and the high-risk group diverged from those in the other two groups at around 800 days is unclear. One possible reason is that the 64 cardiac deaths included 48 deaths from heart failure; therefore, the majority of cardiac deaths were due to heart failure. The worsening of heart failure with age may be one of the reasons for these results.” (page 37, lines 411–416)

*Accordingly, the following references have been removed.*

[23] Tang EW, Wong CK, Herbison P. Global Registry of Acute Coronary Events (GRACE) hospital discharge risk score accurately predicts long-term mortality post acute coronary syndrome. Am Heart J. 2007;153:29-35.

[24] Valgimigli M, Bueno H, Byrne RA, Collet JP, Costa F, Jeppsson A, et al. 2017 ESC focused update on dual antiplatelet therapy in coronary artery disease developed in collaboration with EACTS: The Task Force for dual antiplatelet therapy in coronary artery disease of the European Society of Cardiology (ESC) and of the European Association for Cardio-Thoracic Surgery (EACTS). Eur Heart J. 2018;39:213-60.

[25] Tanaka S, Sakata R, Marui A, Furukawa Y, Kita T, Kimura T. Predicting long-term mortality after first coronary revascularization: – the Kyoto model –. Circ J. 2012;76:328-34.

[26] Nammas W, Kiviniemi T, Schlitt A, Rubboli A, Valencia J, Lip GYH, et al. Value of DAPT score to predict adverse outcome in patients with atrial fibrillation undergoing percutaneous coronary intervention: A post-hoc analysis from the AFCAS registry. Int J Cardiol. 2018;253:35-9.

*We have asked the native English speaker to proofread and have also been modified to make the manuscript easier to understand and to correct any previous grammatical errors as described in “the revised manuscript with track changes”*.

**In summary:**

**- For each paragraph, identify a study implication about why our study is novel, what it adds to the literature, what it suggests PARIS score. Always start with a statement about that implication
- Expand on that implication (ie its application) with evidence for or against it, if it exists
- Close with strong statement about what that implication means in the grand scale of things**

Author’s Reply

*Thank you for putting together your comments. Please see our responses to your comments. Details are given above.*
