# Supplementary material for: Clinical usefulness of the pattern of non-adherence to anti-platelet regimen in stented patients (PARIS) thrombotic risk score to predict long-term all-cause mortality and heart failure hospitalization after percutaneous coronary intervention
Source: PLoS One. 2022 Sep 13;17(9):e0274287. doi: 10.1371/journal.pone.0274287 (PMC9469967; doi:10.1371/journal.pone.0274287)
Supplement: Attachment — Submitted filename: Response to Reviewer R3 2022.8.17.docx [file pone.0274287.s004.docx]

**Responses to Reviewer #2**

We appreciate your detailed review of our manuscript (PONE-D-21-38539). We have revised our manuscript according to your comments.

Reviewer’s comment 1

Thanks for your attempt at fixing the grammar and Discussion section with the help of native English speaker. Unfortunately, I still don't think it is a good discussion that conveys the importance of your findings. I think you need to spend more time showing the feasibility of using your score as it only includes clinical characteristics and remove all the unnecessary details of what the GRACE, TIMI and CADILLAC scores contain (basically remove the second paragraph). You also need to remove strong conclusions such as this: "The PARIS thrombotic risk score can be used to allow for shared decision making and prevent hospitalization due to heart failure pre- and postoperatively. We believe that the results of the current study will help clinicians to identify high-risk patients and to plan early examinations and interventions." & "Therefore, the present study provides a new insight into the clinical usefulness of the PARIS thrombotic risk score" You need to tone down these concluding statements.

Author’s Reply 1

Thank you for your helpful comment. In response to your indication, we have removed the second paragraph of the Discussion section which described details of GRACE, TIMI and CADILLAC scores. Below is the second paragraph that has been removed.

“The GRACE score, which is used for overall risk assessment in patients with ACS, including ST elevation myocardial infarction (STEMI) and non-ST elevation ACS (NSTE-ACS), was designed to calculate the probability of death or myocardial infarction at admission and at six months after undergoing PCI by weighting eight risk factors: age, heart rate, systolic blood pressure, initial serum creatine, Killip class, hospitalization due to cardiac arrest, positive cardiac biomarker, and ST-segment deviation [1-3, 21]. The TIMI risk score, which is often used for risk assessment in patients with NSTE-ACS, is calculated using seven factors: age (≥ 65 years); at least three coronary risk factors (family history, hypertension, dyslipidemia, diabetes mellitus, and smoking); known significant coronary stenosis (≥ 50%); ST changes ≥ 0.5 mm on electrocardiogram; at least two episodes of angina within the previous 24 hours, aspirin use in the past 7 days; and increased cardiac markers. Most of these factors can be assessed immediately after admission. As the score increases, the risk of major cardiovascular complications in the following 2 weeks increases synergistically [4, 21]. The CADILLAC score, which is often used for risk assessment in patients with STEMI, evaluates several different prognostic variables, including LVEF, creatinine clearance, Killip class, final TIMI flow, age, anemia and presence of three-vessel disease. Utilizing the CADILLAC risk score, the patients were stratified into low (CADILLAC score 0–2), intermediate (score 3–5), and high (score ≥ 6) risk groups. The CADILLAC score can be used to accurately predict 30-day and 1-year mortality after primary PCI [5].”

Also, in response to your comment that the tone of the results is too strong, we have revised the sentences in the Discussion section (page 35, lines 366–371 and page 36, lines 383–384), toning them down. The following sentences are the revised sentences.

“Present study has shown the possibility that physicians are able to apply the PARIS thrombotic score not only to estimate the risk of coronary thrombotic events, but also to assess the risk of death and worsening heart failure for patients after PCIs. The PARIS thrombotic risk score may be able to serve as a clinical tool for shared decision making to prevent thrombotic event, hospitalization due to heart failure and death in the clinical practice post PCI.” (page 35, lines 366–371)

“Therefore, the present study suggests that it may provide a new insight into the clinical usefulness of the PARIS thrombotic risk score.” (page 36, lines 383–384)

Reviewer’s comment 2

You need to spend most of the discussion highlighting the importance and feasibility of your score like you did in the third paragraph and end of second paragraph. The connection and flow of ideas between paragraphs need to improve as the last 3 paragraphs tackle completely unrelated ideas. I suggest you show what the score predict (mortality, HF, etc) and why is that important as other scores don't predict all of these, then move to discuss how feasible it is to use the score, and then write on the limitations. Remove paragraph with lines 404-410 as it doesn't add much to the discussion. Keep it simple and straightforward.

Author’s Reply 2

Thank you for your helpful comments and suggestions. In line with your suggestion, we highlighted that the PARIS thrombotic score in the present analysis included not only ACS patients but also patients with chronic coronary syndrome, and could predict the outcomes of death and heart failure hospitalization, which other risk scores do not. We have revised the sentences as below (page 34, line 350 – page 35, line 358).

“The ability of PARIS thrombotic risk score to predict the 6-month mortality of patients with ACS was comparable to that of the GRACE score; hence, we examined the long-term prognostic value of PARIS thrombotic risk score for both ACS and chronic coronary syndrome. In addition, we have shown that the PARIS thrombotic risk score can predict events of heart failure hospitalization that the above scores do not. Heart failure remains a major cause of mortality, morbidity, hospitalization, and poor quality of life in patients with coronary artery disease [21]; thus, identifying patients at risk of heart failure is important to improve their prognosis after PCIs.” (page 34, line 350 – page 35, line 358)

As you pointed out, the last 3 paragraphs of the Discussion section are irrelevant. According to your suggestion, the following 2 paragraph has been removed to keep the discussion simple and straightforward.

“In patients with coronary artery disease after PCIs, various prognostic factors on mortality have been reported previously [27-29]. Co-morbidities such as diabetes mellitus [30], history of acute coronary syndrome, current smoking [31], CKD [32], and history of PCI or CABG [33, 34] have already reported to be risk factors for poor prognosis in patients with coronary artery disease. Since these factors are also components of the PARIS thrombotic risk score, it is possible that this score could be used to predict all-cause death or hospitalization due to heart failure.”

“The reason why the Kaplan-Meier curves for cardiac mortality and hospitalization due to heart failure partially converge and the high-risk group diverged from those in the other two groups at around 800 days is unclear. One possible reason is that the 64 cardiac deaths included 48 deaths from heart failure; therefore, the majority of cardiac deaths were due to heart failure. The worsening of heart failure with age may be one of the reasons for these results.”

We thank you again for your valuable comments on our manuscript.

**Responses to Reviewer #3**

We appreciate your detailed review of our manuscript (PONE-D-21-38539). We have revised our manuscript according to your comments.

Reviewer’s comment 1

Page 4. Study protocol. Overall, 1061 PCI patients were enrolled during a period longer than 8 years. The only exclusion criterium reported in the manuscript was death during hospitalization. On average, about 130 PCI patients per year were enrolled. This mean that the enrolling PCI center was a very low volume hospital (not aligned to the current international standards) and/or in-hospital mortality was very high and/or other exclusion criteria were adopted but not reported. Please review this major issue.

Author’s Reply 1

Thank you for your helpful comment. The present study has enrolled only patients who have undergone their initial PCI at our hospital. We apologize that we missed to write down about this indication. While, our hospital performed about 230 cases of PCI annually between 2010 and 2018 including target lesion or target vessel revascularization and staged PCI after initial one. We think this meets the criteria for adequate institutional volume achieving good outcomes, which is defined as over 200 PCIs per year (J Am Coll Cardiol. 2013; 62: 357-96). We have revised the manuscript about inclusion criteria in the Method section (page 4, lines 79–82). The below is the revised sentences.

“This was an observational study, which enrolled a total of 1,061 consecutive patients who underwent their initial PCI due to stable angina pectoris or acute coronary syndrome (ACS) at Fukushima Medical University Hospital between January 2010 and May 2018.” (page 4, lines 79–82)

Reviewer’s comment 2

Page 5. Study protocol. How was considered deaths from unknow cause? How many deaths from unknown cause were reported?

Author’s Reply 2

Thank you for your helpful comment. Deaths from unknow cause were counted as all cause death. 5 deaths from unknown cause have been reported.

Reviewer’s comment 3

Page 6. Co-morbidities. Dyslipidemia definition is not in line with current guidelines and therapeutic cut-offs. Please review.

Author’s Reply 3

Thank you for your valuable comment. As you mentioned, the definition of dyslipidemia used in the present study is not in line with current guidelines, since we do not know the initial cholesterol levels of patients who were on statins at the time of index PCI. Hence, we inevitably defined dyslipidemia as the recent use of cholesterol-lowering drugs, a triglyceride value of ≥ 150 mg/dL, a low-density lipoprotein (LDL) cholesterol value of ≥ 140 mg/dL, and/or a high-density lipoprotein (HDL) cholesterol value of < 40 mg/dL, as described in the Method section.

Reviewer’s comment 4

Table 1. The use of coronary imaging devices was extremely high (97.2%). Was the study database based on a registry dedicated intracoronary imaging modalities during PCI?

Author’s Reply 4

Thank you for your important comment. This study is not based on any registry dedicated intracoronary imaging modalities. In Japan, intracoronary imaging is accepted in insured medical treatment, so that it is quite usual to use intracoronary imaging devices during daily PCI procedures. Therefore, the use of coronary imaging devices was extremely high.

Reviewer’s comment 5

Tables. The number of Tables and Figures is an issue, and it should be reduced.

Author’s Reply 5

Thank you for your valuable suggestion. As you noted, the number of tables has increased from the first draft. However, due to the meaningful peer review so far, detailed analysis has been performed, and the number of figures and tables has increased. There is no mention of the number of figures and tables in the submission instructions, and we would like to keep the number of figures and tables as they are for better understanding.

Reviewer’s comment 6

Page 36 and 37. Discussion. Please better explain how the PARIS “thrombotic” risk score can predict both the deaths and the hospitalizations for heart failure, the later not always related to a thrombotic event (i.e., but to poor drug adherence). In addition, absence of data regarding long-term pharmacological and intervention treatments should be better discussed in the study limitation section.

Author’s Reply 6

Thank you for your important suggestion. The possible reason why the PARIS thrombotic risk score can also predict all-cause mortality and heart failure due to hospitalization is that major predictors used in the PARIS thrombotic risk score were associated with the risk factors for a poor prognosis of coronary artery disease. It has already been reported that history of acute coronary syndrome and chronic kidney disease is also a risk factor for hospitalization due to heart failure. These explanations were firstly described in the Discussion section, however, the Reviewer 2 recommended us that the above content should not be included in the manuscript because it complicates the content of the discussion section.

While, as you mentioned, the achievement rate of guideline directed medical therapy and the presence or absence of any interventional treatment to the coronary artery disease and the heart failure may affect thrombotic events, death and heart failure hospitalization. We have revised the manuscript and described about this point in the Limitation section (page 36, lines 387–391). Following are the added sentences.

“Second, the clinical practices regarding medical treatment and interventional treatment in the present study may have some difference in the setting of each period. Especially, the achievement rate of guideline directed medical therapy for coronary artery disease and the presence or absence of interventional treatment may affect the rate of thrombotic events, death and heart failure hospitalization after PCI.” (page 36, lines 387–391)

We thank you again for your valuable comments on our manuscript.
